# Coherent changes of wintertime surface air temperatures over North Asia and North America

**DOI:** 10.1038/s41598-018-23750-3

**Published:** 2018-03-29

**Authors:** Bin Yu, Hai Lin

**Affiliations:** 1 Climate Research Division, Environment and Climate Change Canada, Toronto, Canada; 2 Meteorological Research Division, Environment and Climate Change Canada, Dorval, Canada

## Abstract

The surface temperature variance and its potential change with global warming are most prominent in winter over Northern Hemisphere mid-high latitudes. Consistent wintertime surface temperature variability has been observed over large areas in Eurasia and North America on a broad range of time scales. However, it remains a challenge to quantify where and how the coherent change of temperature anomalies occur over the two continents. Here we demonstrate the coherent change of wintertime surface temperature anomalies over North Asia and the central-eastern parts of North America for the period from 1951 to 2015. This is supported by the results from the empirical orthogonal function analysis of surface temperature and temperature trend anomalies over the Northern Hemisphere extratropical lands and the timeseries analysis of the regional averaged temperature anomalies over North Asia and the Great Plains and Great Lakes. The Asian-Bering-North American (ABNA) teleconnection provides a pathway to connect the regional temperature anomalies over the two continents. The ABNA is also responsible for the decadal variation of the temperature relationship between North Asia and North America.

## Introduction

The surface temperature variance and its possible change with global warming are most pronounced in winter over mid-high latitudes^1–4^. Much of the wintertime Northern Hemisphere surface air temperature variability is characterized by the cold ocean-warm land (COWL) pattern^2,3^, which is associated with above-normal heights over Siberia and the Yukon and below-normal heights over the Barents Sea and the North Pacific. However, timeseries of winter surface temperature anomalies with respect to long-term climatological mean over the whole continents of Eurasia and North America are found to be uncorrelated^3^. On the other hand, consistent wintertime temperature anomalies have been observed over large regions in northern Eurasia and North America. In particular, evidence has been presented in the temperature variability ranging broadly from subseasonal to interdecadal time scales^5–10^, the long-term temperature trend^11–14^, and the temperature change with global warming^4,15^. Thus it remains to be quantified where and how the coherent change of temperature anomalies occur over the two continents.

**Figure 1:**
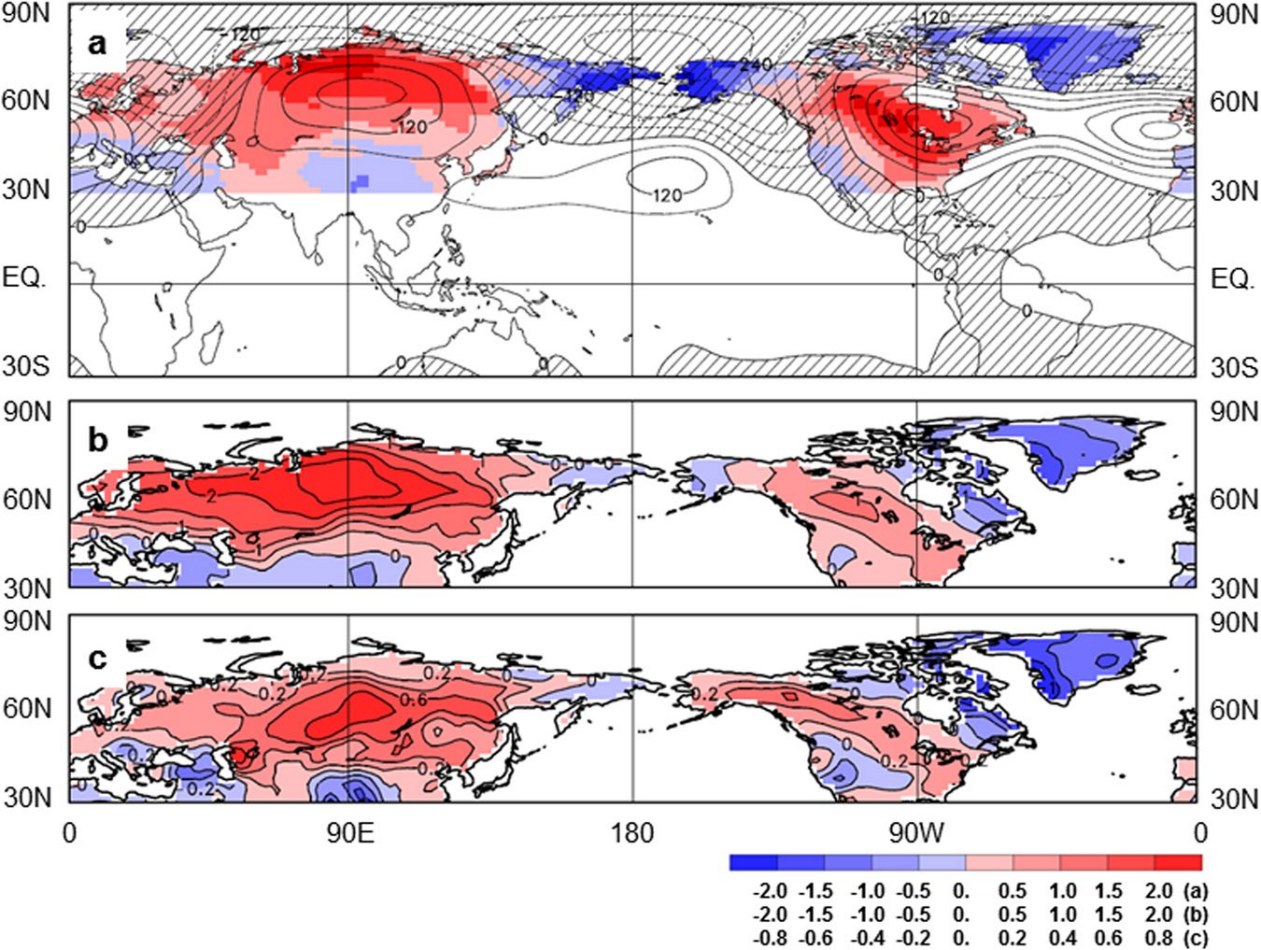
Northern Hemisphere extratropical winter surface temperature anomalies. (**a**) Anomalies of the extratropical T_2m_ (shading, in °C, over lands poleward of 30°N) and the tropical and Northern Hemisphere Φ_500_ (contours, interval 60 m^2^s^−2^) regressed upon the ABNA index from 1951 to 2015. Negative geopotential anomalies are cross-hatched. (**b**) EOF1 of the extratropical T_2m_ anomalies (contours, interval 0.5 °C) from 1951 to 2015. (**c**) EOF1 of the extratropical T_2m_ running 31-yr trends (contours, interval 0.2 °C/decade) for the 35 central years from 1966 to 2000. The central year is the central point of the 31-yr running window. Maps were produced using ECCC/CCCma diagnostics package (http://www.ec.gc.ca/ccmac-cccma).

Recent studies also indicated that, in association with the wintertime warm anomalies over North Asia and the central-eastern parts of North America, the anomalous circulation features a zonally elongated wavetrain originating from North Asia and flowing downstream across Bering Sea and Strait towards North America (Fig. 1a). This reflects an extratropical atmospheric teleconnection, termed the Asian-Bering-North American (ABNA) pattern^7,16^, which is constructed from the geopotential field by excluding the contribution of the tropically originated Pacific-North American pattern^17^. The ABNA teleconnection may connect the temperature anomalies over the two continents. However, whether the ABNA pattern is significantly correlated with the regional temperature anomalies over North Asia and North America and furthermore whether it determines the relationship between the two temperature anomalies remain unclear. These questions are of fundamental importance to climate variability and change and to climate prediction, and are addressed in this study.

## Results

**Figure 2:**
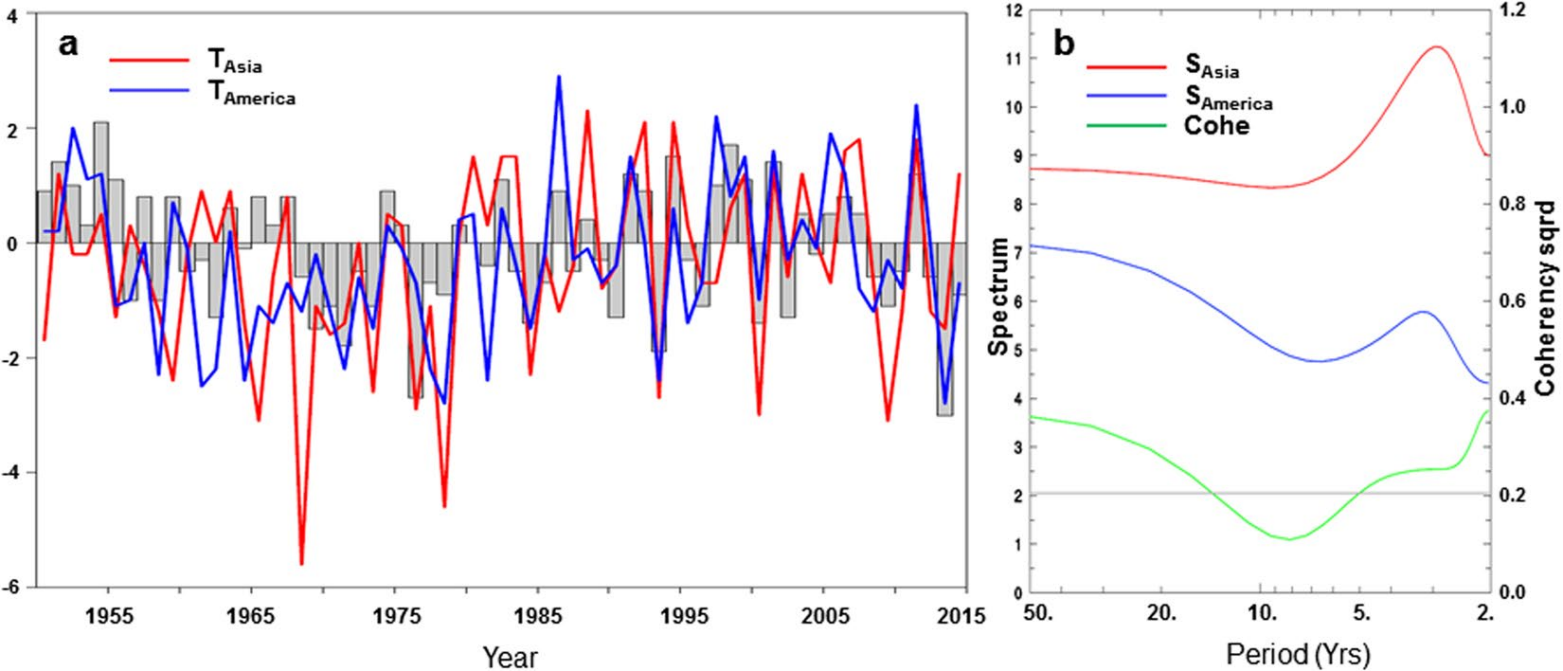
Regional mean temperature anomalies and their spectra. (**a**) Time series of the ABNA index (gray bars, standardized) and the regional mean surface temperature anomalies (°C) over North Asia (red) and North America (blue) from 1951 to 2015. (**b**) Spectrum (red for North Asia and blue for North America) and cross-spectrum (green) of the temperature anomalies. The gray line denotes the 95% confidence level of coherency squared.

The dominant pattern of wintertime Northern Hemisphere surface temperature variability is represented by the leading EOF of the northern extratropical 2 m temperature (T_2m_) anomalies (Fig. 1b, and see methods), which accounts for 27.2% of the total interannual variance and is well separated from subsequent EOFs as per the criterion of ref.^18^. Focusing on the continents of Eurasia and North America, EOF1 is characterized by warm anomalies over North Asia and the central-eastern parts of North America, as well as cold anomalies over subtropical Asia and northeastern Canada, and around Bering Strait. The anomalous temperature pattern bears close resemblance to that associated with ABNA in terms of the spatial structure (Fig. 1a, shading), with spatial correlation of 0.78 over the northern extratropical lands. This indicates that the two temperature patterns have about 60% spatial variance in common over the northern extratropics, and suggests a close relationship between this surface temperature variability pattern and the ABNA atmospheric teleconnection. Two timeseries are created to further characterize the relationship between the temperature anomalies over North Asia (T_Asia_) and North America (T_America_) (Fig. 2a). They are the regional temperature anomalies averaged over the areas defined according to Fig. 1b, with values greater than 1.0 °C over North Asia and 0.5 °C over North America. The areas chosen mainly cover North Asian lands poleward of 40°N and the Great Plains and Great Lakes over North America. However, the results reported here remain virtually unchanged with a reasonable variation of the areas (e.g., using 1.2 °C [1.6 °C] and 0.6 °C [0.8 °C] as the thresholds over North Asia and North America, respectively).

The two regional mean temperature anomaly series are highly correlated, with correlation of 0.43 over the 65 winters from 1951 to 2015 that is statistically significant at the 1% level (see methods). They are also closely correlated with the principal component corresponding to the EOF1 pattern, as would be expected, and with the ABNA index (Table 1). Both T_Asia_ and T_America_ series involve clear interannual and interdecadal variances. A power spectrum analysis further reveals significant power peaks around 3 years for both of them. Consequently, the two regional mean temperature anomaly series co-vary significantly, at the 95% confidence level, over the period from 2–5 years and at interdecadal time scales over 15 years but insignificantly around 10 years (Fig. 2b).

**Table 1:** Correlation coefficients between the regional mean surface temperature anomalies over North Asia (T_Asia_) and North America (T_America_), the ABNA index, and PC1 of the extratropical surface temperature anomalies over the period from 1951 to 2015.

Corr.	T_Asia_	T_America_	ABNA	PC1
T_Asia_	1.0	0.43	0.51	0.91
T_America_		1.0	0.68	0.46
ABNA			1.0	0.47
PC1				1.0

Assuming one degree of freedom per year, the correlation at the 1% significance level is about 0.32 for 65DOF.

**Figure 3:**
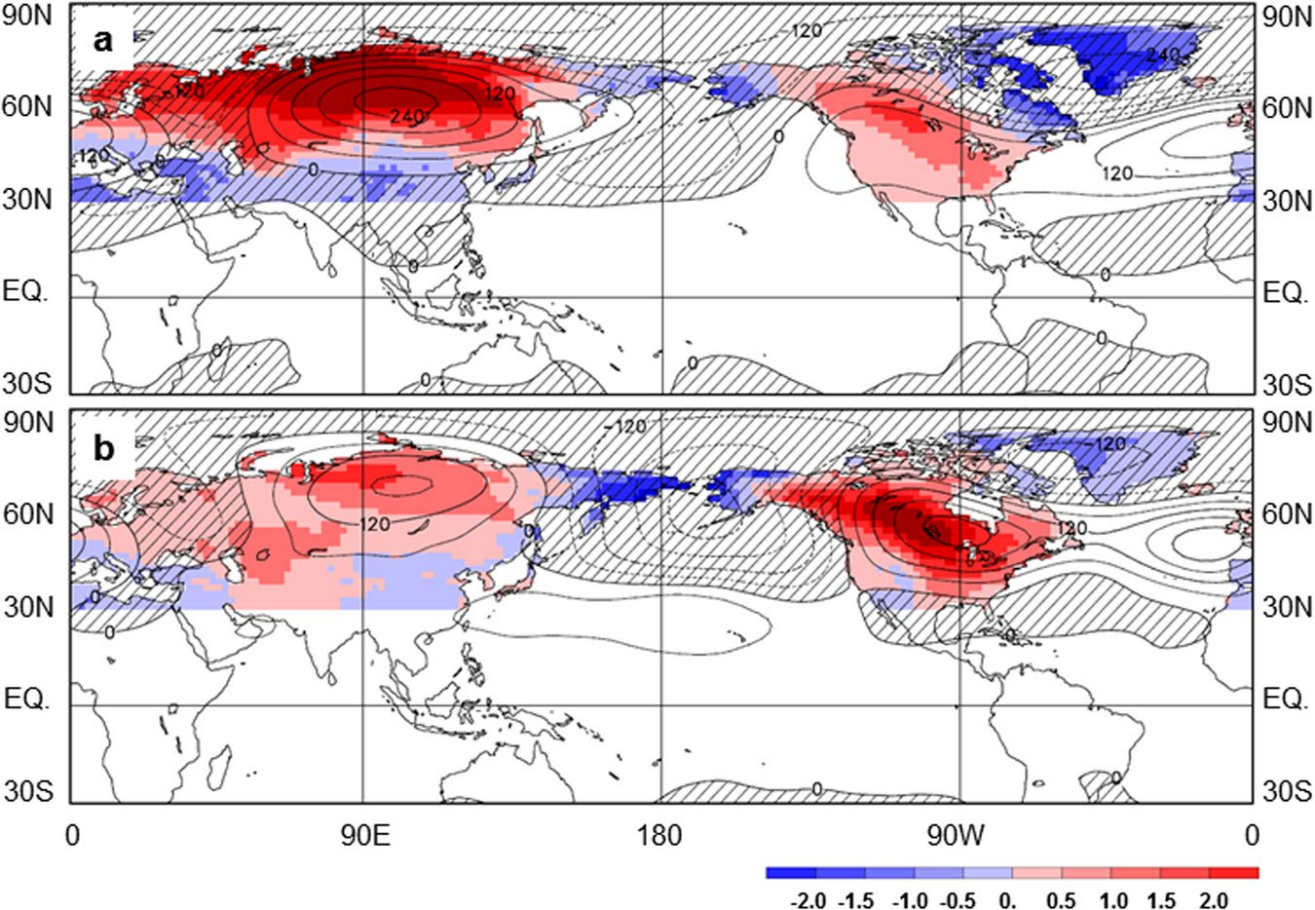
Northern Hemisphere extratropical surface temperature and large-scale circulation anomalies. (**a**) Anomalies of the extratropical T_2m_ (shading, in °C, over lands poleward of 30°N) and the tropical and Northern Hemisphere Φ_500_ (contours, interval 60 m^2^s^−2^) regressed upon the normalized T_Asia_ series from 1951 to 2015. Negative geopotential anomalies are cross-hatched. (**b**) As in (**a**), but for T_2m_ and Φ_500_ anomalies regressed upon the normalized T_America_ series. Maps were produced using ECCC/CCCma diagnostics package (http://www.ec.gc.ca/ccmac-cccma).

Figure 3 displays the anomalies of the northern extratropical T_2m_ and large-scale 500-hPa geopotential (Ф_500_) regressed upon the normalized regional mean temperature anomaly series over North Asia and North America. In association with the positive phase of the T_Asia_ and T_America_ series, T_2m_ is dominated by warm anomalies over North Asia and the central-eastern parts of North America, and cold anomalies around Bering Strait and over subtropical Asia, northeastern Canada and Greenland (Fig. 3, shading). Good correspondence is seen in the two temperature patterns, with differences mainly in the amplitude of the anomalies. The T_Asia_ associated warm anomalies are more pronounced over North Asia and less intense over North America, while the intensity distribution is opposite in the T_America_ associated ones. The anomalous T_2m_ patterns (Fig. 3) also resemble the ABNA associated counterpart (Fig. 1a). The spatial correlation between the ABNA and the T_Asia_ (T_America_) associated temperature anomalies (Figs 1a and 3, shading) is 0.67 (0.88), indicating that the two patterns have about 45% (77%) variance in common over the northern extratropical lands.

Atmospheric circulation may influence the temperature field by advection and through adiabatic processes associated with thermally direct and indirect circulations^3,19^. As can be seen from Fig. 3 (contours), the T_Asia_ and T_America_ associated circulation patterns are dominated by anomalies in the northern extratropics. Above-normal geopotentials at 500-hPa are apparent over north-central Asia and north-central North America, as well as below-normal geopotentials over the Bering Sea and Strait region. This reflects a zonally elongated wavetrain extending from North Asia toward North America, as illustrated also by diagnosis of the stationary wave activity flux (not shown). The anomalous circulation patterns are broadly similar to the ABNA counterpart (Fig. 1a). The spatial correlation between the ABNA and the T_Asia_ (T_America_) associated Ф_500_ patterns (Figs 1a and 3, contours) is 0.65 (0.86) over the northern extratropics, indicating that the two patterns have about 42% (74%) variance in common. The above results demonstrate the coherent change of winter temperature anomalies over the two continents, and suggest that the ABNA teleconnection can be a pathway in linking them.

**Figure 4:**
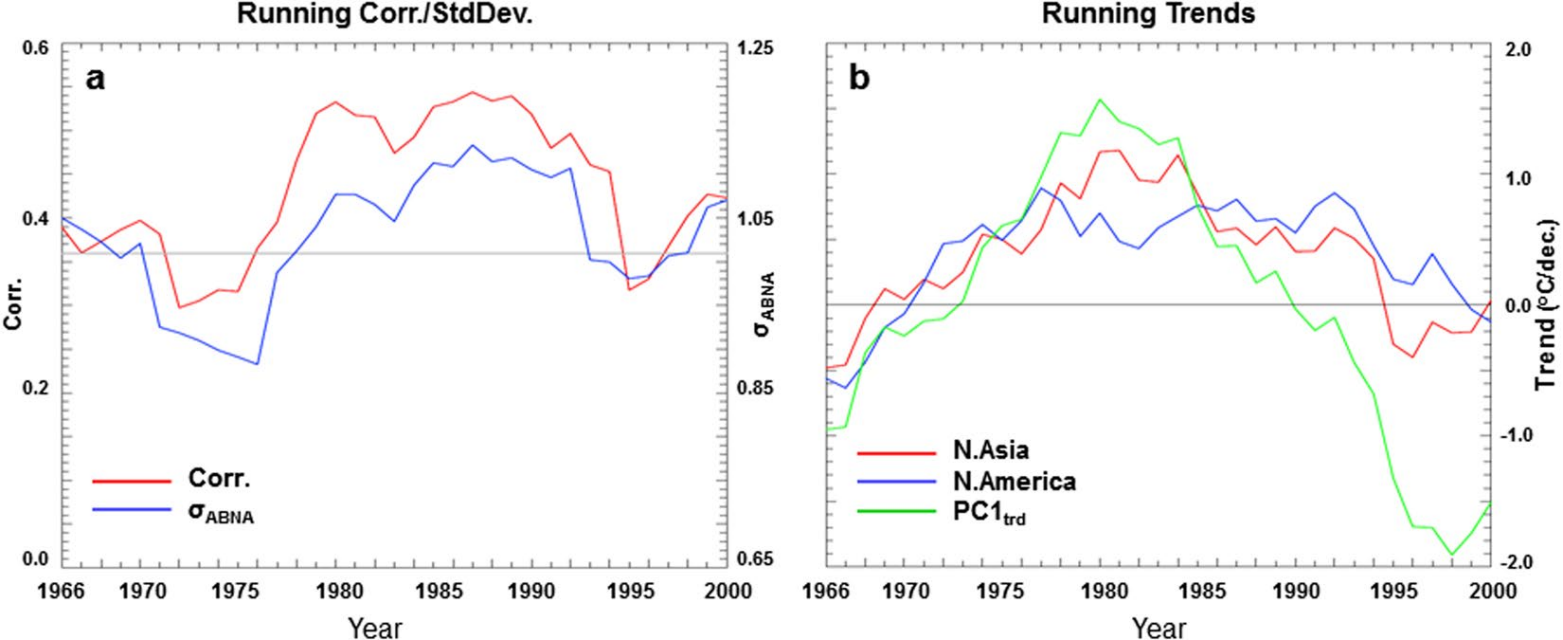
Running correlation, standard deviation, and trends. (**a**) The running 31-yr correlation between T_Asia_ and T_America_ timeseries (red), and the running 31-yr standard deviation of the ABNA index (blue). The gray line denotes the 95% confidence level of correlation. (**b**) The running 31-yr trends of the T_Asia_ (red) and T_America_ (blue) timeseries, and PC1_trd_ of the extratropical T_2m_ trend anomalies (green). Years are labeled based on the central year of the 31-yr window.

The relationship between the temperature anomalies over North Asia and North America is generally steady but with decadal variations. The running 31-yr correlations between the T_Asia_ and T_America_ series range from 0.30 to 0.55, with a mean of 0.43 and high values in the years from 1980 to 1990 (Fig. 4a, red). Here years are labeled on the central point of the 31-yr window. The correlation results depend weakly on the width of the running window (e.g., using 25-yr or 35-yr windows). In addition, the running correlations tend to be consistent with the running variances of the ABNA index (Fig. 4a, blue), with correlation of 0.85 over 1966–2000 between the two evolution curves. This suggests that the ABNA teleconnection variance may be partially responsible for the decadal variation of the relationship between the surface temperature anomalies over North Asia and North America.

T_Asia_ and T_America_ series co-vary at interdecadal time scales. The running 31-yr trends of the two timeseries reveal similar features, generally increased before 1980 and then declined (Fig. 4b, red and blue). Positive trends are seen from 1969–1995 for T_Asia_ and from 1971–1998 for T_America_. The correlation of the two running trend series is 0.75 over the period from 1966–2000, significant at the 1% level, confirming the long-term coherent change of the temperature anomalies over North Asia and North America. The co-varying of temperature trends over the two continents is also evident from an EOF analysis of the extratropical running 31-yr T_2m_ trends for the 35 central years from 1966 to 2000 (Fig. 1c). This EOF1 pattern accounts for 48.6% of the total temperature trend variance and is well separated from subsequent EOFs. Positive anomalies are apparent over North Asia, western Canada, and the Great Plains and Great Lakes in this dominant long-term trend mode. In addition, the pattern somewhat resembles the ABNA associated T_2m_ anomalies and the EOF1 of the extratropical T_2m_ anomalies in terms of the spatial structure (Fig. 1), although the center of action over North Asia shifts slightly southward in the temperature trend pattern compared to that in the temperature variance pattern. This implies the multi-timescale linkage of temperature anomalies over North Asia and North America. The principal component corresponding to the EOF1 trend pattern (PC1_trd_, Fig. 4b, green) also exhibits a changing point around 1980. It is qualitatively similar to the 31-yr running trends of the T_Asia_ and T_America_ timeseries, with correlation of 0.90 (0.61) between PC1_trd_ and T_Asia_ (T_America_) over 1966–2000, significant at the 1% level.

## Summary and Discussion

A coherent variability of winter surface temperature anomalies is found over North Asia and the central-eastern parts of North America, especially the Great Plains and Great Lakes. This is supported by the EOF analysis of the northern extratropical surface temperature and temperature trend anomalies and the timeseries analysis of the regional averaged temperatures over North Asia and the Great Plains and Great Lakes. The temperature anomalies over the two regions are correlated significantly at interannual time scales from 2–5 years and interdecadal scales over 15 years. The ABNA teleconnection plays an important role in linking the temperature anomalies over the two continents. In addition, the ABNA is partially responsible for the decadal variation of the temperature relationship between North Asia and North America.

**Figure 5:**
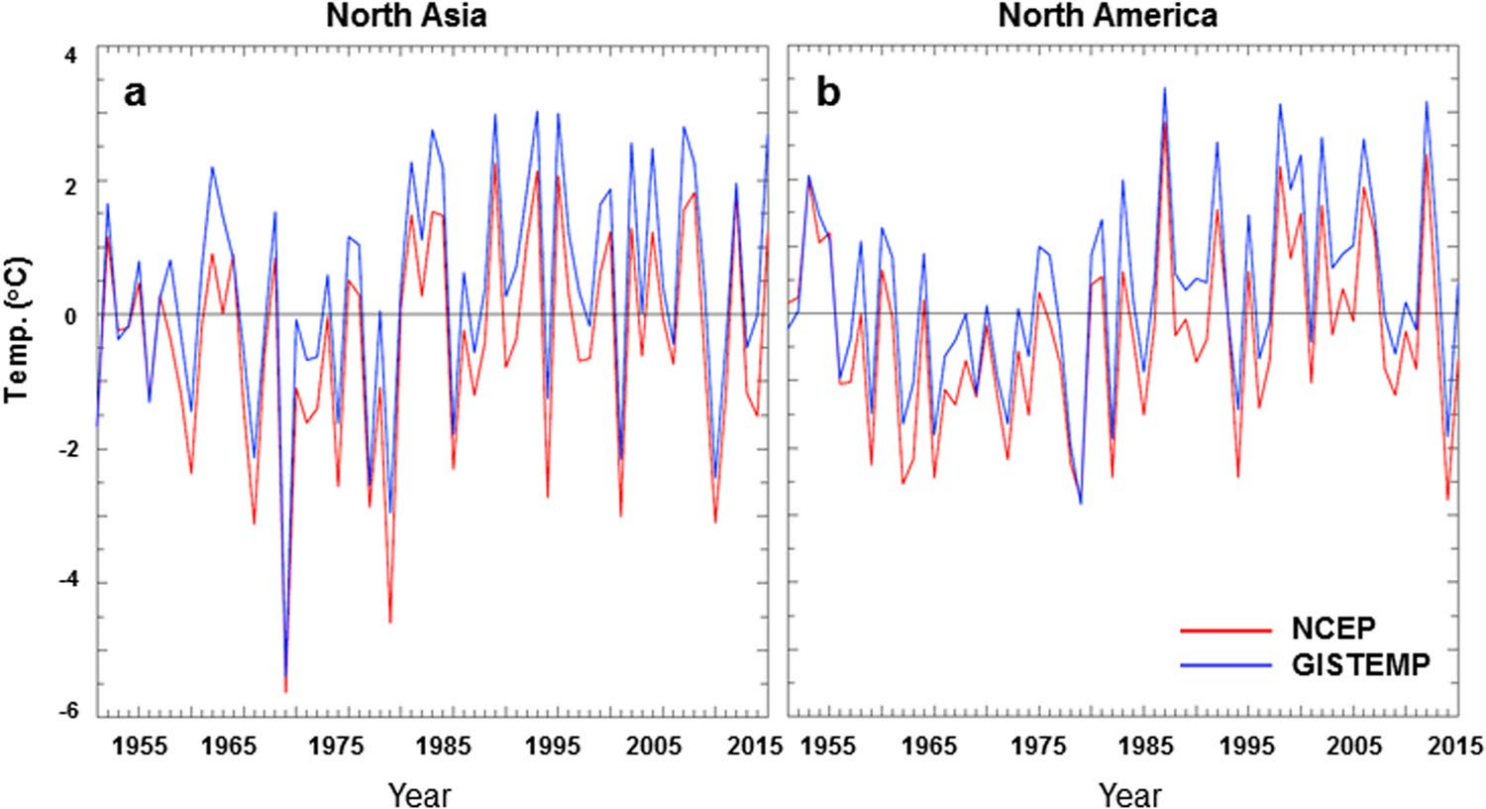
Regional mean temperature anomalies. Time series of the regional mean surface temperature anomalies over North Asia (left) and North America (right) for the NCEP reanalysis (red) and the GISTEMP analysis (blue) from 1951 to 2015.

The temperature relationship between North Asia and North America is confirmed by analyzing the observation based GISTEMP data (see methods). The striking similarity of the regional averaged temperature anomalies with respect to the 1951–2015 climatology is seen from the NCEP and GISTEMP datasets (Fig. 5), with correlations of about 0.98 for both series over the 65 winters, significant at the 1% level. Nevertheless, the regional mean temperature anomalies in North Asia (North America) are slightly higher in the GISTEMP analysis than in the NCEP reanalysis, with a mean of 0.76 °C (0.65 °C). In addition, several relevant questions merit further study. In particular, does the winter temperature relationship between North Asia and North America appear in other seasons and also connect by the ABNA teleconnection? Are there any lag correlations of subseasonal and seasonal temperature anomalies between the two continents, which have important implications for improving climate predictions on subseasonal-interannual time scales?

The ABNA and COWL patterns contain similar elements over North Asia and North America, and differences are mainly over the oceans^16^. In association with the positive phase of the two patterns, the ABNA is characterized by below-normal heights over the Bering Sea and Strait region as well as relatively weak above-normal heights over the North Pacific, while the COWL is marked by below-normal heights over the Barents Sea and the North Pacific. The winter surface temperature anomalies associated with ABNA also bear some resemblance to those related to the Arctic Oscillation (AO)^20^, especially over North Asia. However, pronounced warm anomalies in association with the positive ABNA phase are seen over the entire central-eastern region of North America, especially the Great Plains and Great Lakes, while warm anomalies only appear over part of central and eastern North America for the AO counterpart^11,14^. Nevertheless, it should be noted that the coherent change in the Northern Hemisphere extratropics may also arise through other processes, such as the dynamical pathway involving the stratosphere, the Eurasian snow cover, and their relationship to the AO^21,22^, the positive feedback between winter blocking activities and the northern land-sea thermal contrast^23^, and the Pacific Decadal Oscillation^24–27^. In addition, it remains to be explored the coherent change of surface temperatures over the two continents with external forcings, such as energy consumption and anthropogenic greenhouse gas emissions^28,29^.

## Methods

The analysis is based on December-February (DJF) mean surface air temperature and 500-hPa geopotential data obtained from the National Centers for Environmental Prediction (NCEP)/National Center for Atmospheric Research reanalysis^30^ and the National Aeronautics and Space Administration Goddard Institute for Space Studies surface temperature analysis (GISTEMP)^31^ (surface air temperature with 1200 km smoothing, https://data.giss.nasa.gov/gistemp) from 1951 to 2015. Years refer to the January dates. The variables are analyzed on standard 2.5° × 2.5° grids. The PNA index, identified from the rotated empirical orthogonal function (REOF) analysis of monthly mean standardized 500-hPa height anomalies over the northern extratropics, for the same period was downloaded from the Climate Prediction Center (http://www.cpc.ncep.noaa.gov/data/indices).

The ABNA teleconnection index (Fig. 2a, gray bars) is constructed from the standardized 500-hPa geopotential field by excluding the PNA pattern contribution, following refs^7,16^. The relationship between a timeseries of interest and its associated variables is quantified through regression and correlation analyses. The Student t test is used to assess whether the correlation between two timeseries is statistically significant, assuming one degree of freedom (DOF) per DJF. Similar significant results are obtained using the effective sample size estimated by considering the autocorrelation of the timeseries of interest^32^. In addition, the empirical orthogonal function (EOF) analysis is employed to identify the principal mode of surface temperature and temperature trend anomalies over the northern extratropics. The power spectra and cross-spectra of timeseries are calculated using the Parzen estimator^33^. Cross-spectrum analysis allows one to determine the relationship between two timeseries as a function of frequency.

### Code availability

The code associated with this paper is available on request from B.Y.
